# An AI-Based Telerehabilitation Solution to Improve Mobility in People With Multiple Sclerosis (the PLATINUMS Project): Protocol for an Implementation and Evaluation Study

**DOI:** 10.2196/75983

**Published:** 2025-07-24

**Authors:** Lars Hvid, Susan Coote, Massimiliano Pau, Alon Kalron

**Affiliations:** 1 Exercise Biology Department of Public Health Aarhus University Aarhus Denmark; 2 The Danish Multiple Sclerosis Hospitals Ry and Haslev Denmark; 3 Physical Activity for Health Research Centre University of Limerick Limerick Ireland; 4 Multiple Sclerosis Society of Ireland Dublin Ireland; 5 Department of Mechanical, Chemical, and Materials Engineering University of Cagliari Cagliari Italy; 6 Department of Physical Therapy, Stanley Steyer School of Health Professions Gray Faculty of Medical and Health Sciences Tel Aviv University Tel Aviv Israel; 7 Multiple Sclerosis Center Sheba Medical Center Tel-Hashomer Israel

**Keywords:** multiple sclerosis, telerehabilitation, mobility, feasibility study, randomized controlled trial, remote exercise, validation, cost-effectiveness

## Abstract

**Background:**

Multiple sclerosis (MS) is a chronic, progressive, and neurodegenerative disease affecting more than 2.8 million people globally. Mobility impairments are among the most significant challenges faced by people with MS, leading to physical inactivity, deconditioning, and disability progression (for some, even irreversible disability). This negatively impacts mental health, social participation, and quality of life while placing a considerable economic burden on society. Exercise can improve mobility and mitigate disability progression, but facility-based options are often inaccessible, especially for those in remote areas. Telerehabilitation offers a promising alternative, but current systems are limited by complexity and hardware requirements.

**Objective:**

The PLATINUMS (Implementation of an Advanced Telerehabilitation Solution for People With Multiple Sclerosis) project proposes an AI-driven telerehabilitation system to deliver accessible, cost-effective, and home-based exercise therapy for people with MS.

**Methods:**

The PLATINUMS project begins with working package (WP) 1, focusing on obtaining ethical approval and recruiting staff. Following this, WP2 involves a 4-week system feasibility and usability study (n=40) to assess and refine the digital platform. WP3 comprises a validity study (n=60) to evaluate remote mobility tests via the system, such as the Short Physical Performance Battery (SPPB), functional reach, and sit-to-stand tests, to ensure their reliability for use in WP4, the feasibility multicenter randomized controlled trial (RCT). The 10-week multicenter feasibility RCT will be conducted in MS centers across Denmark, Ireland, Israel, and Italy, with 96 participants varying in disability levels. The primary objective is to evaluate the efficacy of the AI-powered telerehabilitation system on mobility outcomes compared to usual care. Finally, WP5 will assess the cost-effectiveness of the telerehabilitation system by analyzing implementation costs, adherence, and use of health care. The PLATINUMS project aims to revolutionize exercise therapy for people with MS by demonstrating the feasibility, validity, and preliminary efficacy of the AI-driven telerehabilitation system. This approach addresses barriers such as accessibility, privacy, and standardization while promoting patient and therapist acceptance.

**Results:**

Funding for the PLATINUMS project was obtained in February 2024. WP2 data collection began in April 2025 across 4 European sites. WP3 is scheduled to launch in July 2025, with WP4 (the feasibility RCT) planned for January 2026. Initial WP2 results are expected by October 2025, with first publications anticipated in mid-2026.

**Conclusions:**

The PLATINUMS project is expected to generate critical insights into the feasibility, usability, and preliminary efficacy of an AI-based telerehabilitation system for people with MS. By leveraging widely available technology and real-time feedback, the system addresses key barriers to traditional rehabilitation. Findings from this protocol may inform future large-scale trials and support the broader implementation of digital health solutions in neurological rehabilitation.

**International Registered Report Identifier (IRRID):**

PRR1-10.2196/75983

## Introduction

Multiple sclerosis (MS) is a chronic and progressive disease of the central nervous system that affects more than 2.8 million people worldwide (35.9 per 100,000 people) [1]. One of the most common problems reported by people with MS is impaired mobility, specifically the inability to walk safely and independently [2,3]. Approximately 85% to 90% of people with MS report difficulties with mobility during the disease course [4], and these difficulties can commence very early after onset [5,6]. Due to progression of the disease, most people with MS will eventually require a walking aid (ie, a cane or walker) [7], and in some more severe cases, mobility is possible only by using a wheelchair or scooter [8]. Decreased mobility is, in part, also caused by reductions in physical activity [9], likely mediated through deconditioning of different physiological systems (eg, the cardiorespiratory system) [10]. At some critical points, mobility is limited to such an extent that there is an irreversible disability, resulting in secondary health conditions that are difficult, if not impossible, to treat [11]. This progression of disability also impacts mental health, which further contributes to decreased social, recreational, and vocational participation in society and ultimately results in a poor quality of life [12]. The decrease in participation and work productivity, in combination with an increase in secondary health conditions and medical needs, significantly impacts the global economy worldwide [13,14]. Since MS is the leading cause of disability in young and middle-aged adults, maintaining mobility is essential. Therefore, measures to combat or reverse this downward spiral and progression of disability must be developed and implemented to reduce the burden of this disease.

Exercise can improve mobility and perhaps decrease the rate and extent of disability (ie, impaired physical function) in people with MS [15,16]. Exercise programs based in hospitals and clinical facilities, particularly among other people with MS or under the supervision of MS experts, may help people with MS participate in exercise or exercise at a higher, more intense level [17]. However, a lack of access to these programs, especially for those who live in remote areas where there are few options or where there are health care and medical facilities but no MS experts, may make it difficult to engage in facility-based exercise training programs [18-20]. The COVID-19 pandemic highlighted the need to find alternative solutions to increase exercise and physical activity in the population with MS. In a series of studies involving members of the present consortium, we reported that physical activity and exercise activities, especially at moderate and high intensities, decreased during the pandemic in people with MS [21,22]. Moreover, the research consortium has strongly concluded that to ensure the continuity of exercise training for people with MS during future pandemics, health care stakeholders should be highly encouraged to provide training via advanced remote technologies concurrently with professional guidance and support [23].

Telerehabilitation has excellent potential for providing accessible and cost-effective options for exercise in the home environment for people with MS, particularly if there is ongoing communication with knowledgeable exercise personnel who ensure that exercise is carried out safely and optimally (ie, required movements are performed correctly). However, only a few studies have tested the capability of telerehabilitation to increase exercise and physical activity participation in people with MS, with some encouraging results [24-26]. Nevertheless, despite the advantages associated with the use of telerehabilitation, several limitations still exist that prevent the use of this technology on a large scale. In particular, most commercially available solutions do not provide feedback when the patient does not perform the exercise correctly or require special hardware (eg, movement sensors) that can be hard to set up for nonexpert users [27].

In this project, we intend to implement cutting-edge artificial intelligence (AI) technology designed by WizeCare [28] for a home-based exercise therapy program for people with MS. One of the advantages of this system is its technical simplicity. The system requires only a mobile phone, tablet, or personal computer and has no need for additional third-party hardware. This feature is essential for people with MS with low digital literacy [29]. Moreover, the technology incorporates an AI algorithm that calculates the range of motion and movement speed based on acquired videos of the user performing a specific exercise, enabling real-time feedback and positive encouragement during practice. Lastly, the system includes over 800 exercise drills classified according to targeted body regions and aspects of treatment (ie, flexibility, strength, or coordination) that the therapist can customize to create a personalized exercise program according to each patient’s goals and needs. Notably, the telerehabilitation system has been implemented in numerous health care services with thousands of users (mainly in orthopedic rehabilitation) and has yielded encouraging results in conjunction with positive feedback from patients, physical therapists, and stakeholders. Nonetheless, it has never been systematically tested in the population with MS.

Therefore, our overall goal is to implement an innovative telerehabilitation exercise training system for people with MS by assessing its efficacy in improving mobility. For this primary aim, we propose a pilot multicenter randomized controlled trial (RCT) in established MS centers across the European Union community in Italy, Ireland, and Denmark, as well as in Israel, to establish trial feasibility and to compare exercise delivery via telehealth with usual care. Further innovative aims of the project include (1) assessing the impact of a telerehabilitation exercise training program on mood and quality of life; (2) validating a set of remote mobility clinical tests delivered via the telerehabilitation system to evaluate balance and functional mobility in people with MS; and (3) examining the cost-effectiveness and value of the telerehabilitation exercise intervention program for people with MS. This information is scarce in the treatment of people with MS.

Ultimately, the knowledge obtained from this research will be relevant not only for people with MS; it is expected to also be of great value in understanding the most helpful way to deliver the benefits of home-based exercise therapy to other vulnerable populations, including many older adults who may appear healthy but are not, and others with neurological impairments (eg, stroke or Parkinson disease).

## Methods

### Overview

**Figure 1:**
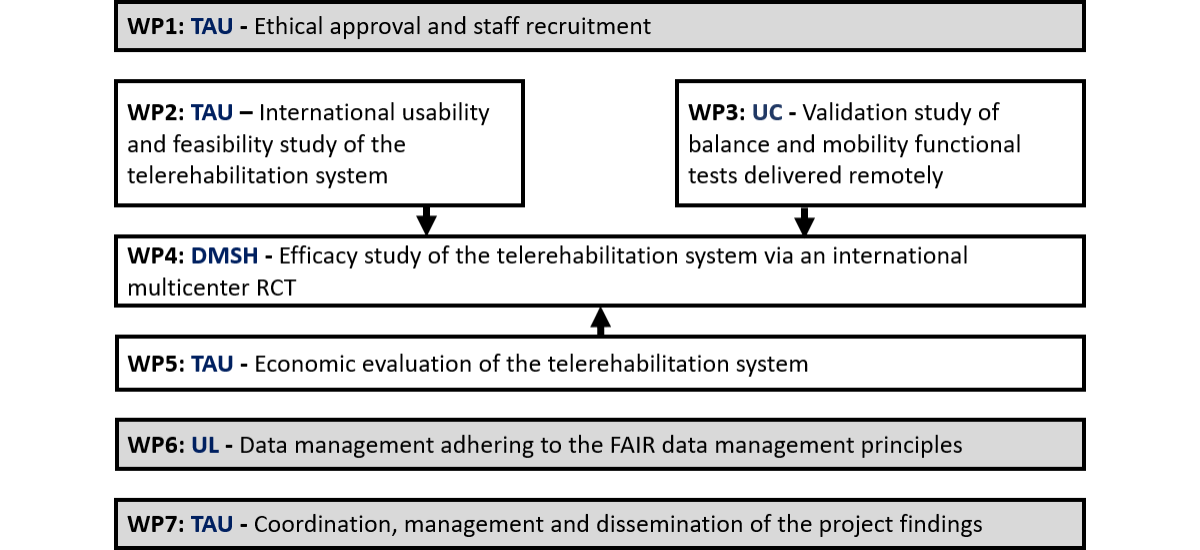
PLATINUMS (Implementation of an Advanced Telerehabilitation Solution for People With Multiple Sclerosis) project overview based on working packages (WPs). DMSH: Danish Multiple Sclerosis Hospitals; FAIR: findable, accessible, interoperable, and reusable; RCT: randomized controlled trial; TAU: Tel Aviv University; UC: University of Cagliari; UL: University of Limerick.

Our main objective is to collect evidence to facilitate the implementation of an innovative AI telerehabilitation exercise training system in MS clinical practice. After the completion of working package (WP) 1, which focuses on obtaining ethical approval and recruiting staff, our first objective (WP2) will be to investigate the usability and feasibility of the WizeCare system in MS centers in 4 European countries, encompassing a range of people with MS with varying disabilities and demographics. The second objective (WP3) will be to develop and psychometrically test a range of mobility measures collected by the telehealth system. The third objective (WP4) will be to compare exercise delivered via telehealth with usual care. The primary outcome will be trial feasibility, with preliminary efficacy for mobility, mental health, social participation, and quality of life outcomes. The fourth objective (WP5) will be to conduct an economic evaluation of exercise program delivery via telehealth compared with in-person care. The final objective will be to work collaboratively with clinicians, policymakers, and people with MS and to widely disseminate the knowledge generated by this program to optimize implementation. Figure 1 presents the WPs of the PLATINUMS (Implementation of an Advanced Telerehabilitation Solution for People With Multiple Sclerosis) project, while Table 1 illustrates its expected timeframe.

**Table 1:** PLATINUMS (Implementation of an Advanced Telerehabilitation Solution for People With Multiple Sclerosis) Gantt chart describing each working package (WP) and task (T).

		Year 1					Year 2					Year 3			
		Q1	Q2	Q3	Q4	Q5		Q6	Q7	Q8	Q9		Q10	Q11	Q12
**WP1: Ethical approval, staff recruitment**		✓													
**WP2: International usability and feasibility study of the telerehabilitation system**			✓	✓	✓	✓									
	T2.1. Installation of the telerehabilitation system, including private data security and cloud data management		✓												
	T2.2. Harmonization between staff members operating the telerehabilitation system and intervention program		✓												
	T2.3. Testing the system on a small group of participants for feasibility (patient/therapist), integrity, security, and proper transfer of data			✓	✓	✓									
**WP3: Development and validation study of balance and mobility functional tests delivered remotely**			✓	✓	✓	✓		✓							
	T3.1. Development of mobility assessment tests to be delivered remotely via the telerehabilitation system		✓	✓											
	T3.2. Establishing reliability and validity of the remote mobility tests			✓	✓	✓		✓							
	T3.3. Developing standardized instructions and training materials							✓							
**WP4: Efficacy study of the telerehabilitation study via a pilot multicenter RCT^a^**								✓	✓	✓	✓		✓	✓	
	T4.1. Coordination between staff members operating the intervention programs and blind assessors on all procedures related with the outcome measures							✓							
	T4.2. Implementation of intervention programs							✓	✓	✓	✓		✓		
	T4.4. Data analysis of remote and clinical outcome measures												✓	✓	
	T4.5. Assessing adherence to and satisfaction with (among participants/therapists) the telerehabilitation intervention program												✓	✓	
**WP5: Economic evaluation of the telerehabilitation system**								✓	✓	✓	✓		✓	✓	
	T5.1. Identifying the perspective of the economic evaluation and finalizing the specific study questions in terms of economics							✓							
	T5.2. Identifying all the costs associated with the implementation of the telerehabilitation system and intervention program							✓							
	T5.3. Following costs of the RCT and identifying all the benefits of the telerehabilitation system and intervention program							✓	✓	✓	✓		✓		
	T5.4. Cost-effectiveness (cost-utility) analyses													✓	
	TS.5. Interpretation and reporting of results														✓
**WP6: Data management adhering to the FAIR^b^ data management principles**		✓	✓	✓	✓	✓		✓	✓	✓	✓		✓	✓	✓
**WP7: Coordination, management, and dissemination**		✓	✓	✓	✓	✓		✓	✓	✓	✓		✓	✓	✓
	17.1. Dissemination of project findings								✓	✓	✓		✓	✓	✓
Meetings		F^c^	O^d^	O	O	O		F	O	O	O		O	O	F

^a^RCT: randomized controlled trial.

^b^FAIR: findable, accessible, interoperable, and reusable.

^c^F: face-to-face.

^d^O: online.

### Research and Innovation Questions

This innovative project will include 5 key research questions (RQs), which we propose will fill in the gaps in our current knowledge.

RQ1 (WP2) is as follows: In people with MS, what is the usability and feasibility of an advanced telerehabilitation system in facilitating remote exercise training? Qualitative and quantitative measures will be used to evaluate the usability and feasibility of the telerehabilitation system in 4 countries. Following necessary system upgrades and adaptations, findings will be incorporated into the feasibility multicenter RCT (WP4). Based on data from other patient populations, we hypothesize that the innovative telerehabilitation system will score highly for usability and feasibility among the participants with MS.

RQ2 (WP3) is as follows: In people with MS, are scores provided by clinical mobility tests obtained via tele-rehabilitation valid and reliable? Evidence for remote mobility evaluation does not currently exist for people with MS. We will evaluate 5 tests developed from commonly used clinical measures. We hypothesize that innovative remote mobility tests will demonstrate good-to-excellent validity and reliability.

RQ3 (WP4) is as follows: In people with MS, is a large multicenter RCT possible, and can it show preliminary efficacy of an individualized exercise program delivered via telerehabilitation to improve mobility compared with in-person physical therapy? An international pragmatic multicenter feasibility RCT will be conducted to compare the effectiveness of delivering the individual exercise program via telerehabilitation versus in person by a physiotherapist. We hypothesize that both exercise intervention programs will improve mobility in people with MS; however, nonsignificant differences in mobility outcome measures between groups (telerehabilitation vs usual care) will be demonstrated.

RQ4 (WP4) is as follows: In people with MS, does an individualized exercise program delivered via telerehabilitation have a comparable effect on mood and quality of life (QoL) compared with standard physical therapy (facility based)? This RQ will be answered in the planned international multicenter feasibility RCT, preceded by a feasibility and usability study. Consequently, our hypothesis is that mood and QoL will be improved in participants whose symptoms are significant at baseline.

RQ5 (WP5) is as follows: In people with MS, does a personalized exercise program delivered via telerehabilitation yield comparable cost-effectiveness value compared with standard physical therapy (facility based)? We hypothesize that the telerehabilitation system will be more cost-effective than personal exercise provided by a physiotherapist. We will account for several potential confounders, such as (1) disability, (2) geographic location, and (3) comorbidities.

### Methodology, Approach, and Study Population

The different WPs of the research project and timeline are presented in Figure 1 and Table 1. The first phase of this project will examine the system usability and feasibility of an innovative advanced telerehabilitation system in people with MS (WP2). The system usability and feasibility study will be conducted in each participating country (Israel, Italy, Ireland, and Denmark) with a small group of people with MS (n=10 per center) with varying disability. Participants will be recruited according to the following criteria: aged 18 years or older; confirmed diagnosis of MS based on the McDonald criteria [30] (both relapsing-remitting and progressive MS); an Expanded Disability Status Scale (EDSS) score of 2.5-6.5; willing to travel to the local study site for the evaluation sessions; and internet service, and having a device (eg, smartphone, tablet, or PC) equipped with a camera that meets the technical specifications recommended by the telerehabilitation system manufacturer. Individuals who have had a documented relapse during the past 3 months will be excluded, as will those who are unable to follow simple instructions.

Following assessment and exercise prescription, participants will complete an exercise program delivered by the telerehabilitation system. The short-term program will include 8 exercise training sessions (45- to 60-minute sessions twice weekly for 4 weeks). The amount of training throughout the short-term program will be sufficient for collecting data and taking note of the safety, usability, and feasibility of the advanced rehabilitation technologies [31]. The primary outcome measure of WP2 will be collected at the end of the short-term telerehabilitation exercise program with the Telehealth Usability Questionnaire (TUQ) [32], a solid, robust, and versatile tool used to measure the quality of the telehealth system’s interaction and services. In addition, qualitative data will be collected through semistructured interviews with key stakeholders (people with MS and therapists) and analyzed thematically.

In parallel with the system feasibility and usability study, we plan to develop and psychometrically test a set of remote balance and functional mobility tests delivered via the telerehabilitation system (WP3). We will adapt and manualize 5 remote mobility tests that are collected through the telerehabilitation system: the 30-second sit-to-stand (30STS) test, timed-up-and-go (TUG) test, functional reach test (FRT), 20-second single-leg stance (20SLS) test, and the Short Physical Performance Battery (SPPB). The SPPB includes the following tests: side-by-side stand (10 seconds), semitandem stand (10 seconds), tandem stand (10 seconds), a gait speed test over 3 meters, and a 5-time repeated chair stand test. Upon completion of the developmental phase, we will conduct a test-retest reliability study for in-person and telehealth data capture and a criterion validity study comparing telehealth and in-person data with 15 people with MS (EDSS 2.5-6.5) at each site (n=60).

To examine the efficacy of the advanced telerehabilitation solution for people with MS, we propose an international feasibility multicenter RCT (WP4). We will include a target sample of 24 participants per country (in 4 countries; 96 participants in total) based on the stepped rule of thumb proposed by Whitehead et al [33], who estimated the optimal sample size for an early-stage/pilot RCT to be 24. Importantly, as the results may differ across countries, we consider each country an individual site for the feasibility RCT. Statistical analysis will be performed for the total group and each country separately. The participants will be randomly allocated (individual randomization) into one of two groups: (1) home-based telerehabilitation exercise training via the WizeCare platform [28] or (2) usual care. The sample criteria for the feasibility multicenter RCT will be the same as those for WP2.

### Intervention and Comparators

**Figure 2:**
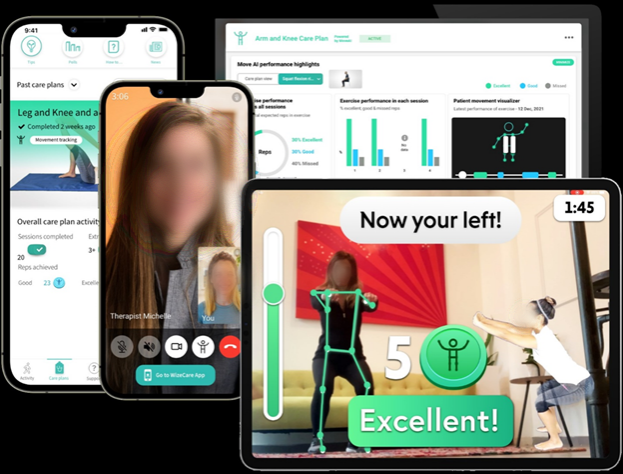
Key elements of the telerehabilitation digital platform.

As previously mentioned, the telerehabilitation intervention program will be delivered through the AI telerehabilitation platform designed and marketed by WizeCare [28]. WizeCare is a medium-sized digital health company that has developed an innovative solution for remote physical therapy and exercise training at home. The system includes over 800 exercises that encompass strength, coordination, and flexibility, targeting the lower and upper body and core muscle groups and joints, enabling the therapist to create a tailored exercise program. Moreover, the system includes a technology called MoveAI (patent no. US 11,636,777 B2), which identifies, tracks, and analyzes the user’s movement without requiring customized sensors but instead using the camera available on a smartphone, tablet, or PC. This will provide the user (ie, patient) with immediate feedback and guidance. Figure 2 illustrates key features of the telerehabilitation digital platform. The system is safe and has been confirmed by several security certificates of the highest standard (ISO/IEC 27001:2013; ISO27799:2016; AMAR [Israeli standard]: 29460001). Additional data on adherence and compliance will be collected via the WizeCare telerehabilitation platform.

The telerehabilitation exercise intervention program will comprise a 10-week, physiotherapist-prescribed, evidence-based, individualized exercise program based on the exercise guidelines for people with MS and the physiotherapist’s clinical reasoning [34]. Eligible participants will receive tailored exercise instructions, which will include performing 8 to 10 exercises in accordance with their abilities and functional needs. Following the first week of training, the participant will be contacted by a physical therapist (via video or audio chat through the system) to monitor participation, progress, and adverse events. Participants in the telerehabilitation group will be compared to those in usual care. Since the term “usual care” is broad, particularly across countries, and physical activity participation varies, participants in both groups will provide weekly information about their physical activity based on the FITT-VP (frequency, intensity, time, type, volume, and progression) principles [35]. To measure adherence, all participants will receive a diary and be requested to log their exercises and submit reports weekly. The report submission will be monitored weekly with a phone call by a physical therapist.

### Outcome Measures for the Feasibility Multicenter RCT

The outcome measures for WP4 will include the 30STS [36] (primary outcome), SPPB [37], 6-minute walk test (6MWT) [38], timed 25-foot walk test (T25FWT) [39], mini-BESTest (balance) [40], FRT [41], and a self-report questionnaire, the 12-item MS Walking Scale (MSWS-12) [42]. In accordance with the project’s secondary aims, we will examine the impact of the intervention programs on mood (ie, depression and anxiety with the State-Trait Anxiety Inventory [STAI] [43] and Quick Inventory of Depression Symptomology [QIDS] [44]), quality of life (EQ-5D-5L [45]) and the impact of MS (29-question Multiple Sclerosis Impact Scale; MSIS-29 [46]).

All outcome measures will be collected at baseline (at the initiation of the intervention program), and after the intervention period’s completion. Assessors (physical therapists) will be blinded to group allocation, and patient report outcomes will be collected using an online form. Other significant disease-related factors collected solely at baseline (together with demographic and relevant clinical information) will include perceived fatigue (Modified Fatigue Impact Scale; MFIS [47]) and cognition (Symbol Digit Modality Test; SDMT [48]). Additionally, we will collect baseline information on digital literacy, which may confound the intervention’s effectiveness. All outcome measures used for the study have been validated in people with MS and are recommended for rehabilitation and clinical trials.

### Data Analysis and Statistical Plan

Each research question is addressed through specific outcome measures. For RQ1, usability and feasibility will be assessed via the TUQ, adherence rates, dropout rates, and stakeholder interviews, with qualitative data analyzed thematically. RQ2 involves psychometric evaluation of remote assessments (e.g., SPPB, STS, and TUG) using intraclass correlation coefficients for test-retest reliability and Pearson correlations to compare remote and in-person scores. RQ3 and RQ4 outcomes (eg, 30STS, MSWS-12, EQ-5D-5L, STAI, and QIDS) will be analyzed using linear mixed models to assess within- and between-group effects over time. The primary analysis will follow an intention-to-treat principle, including all randomized participants in the groups to which they were originally assigned, regardless of adherence to the intervention protocol. A per-protocol analysis may be conducted as a secondary exploratory analysis. Missing data will be addressed using multiple imputation, assuming data are missing at random, and sensitivity analyses will be performed to assess the robustness of results.

As part of RQ5, the study also incorporates a comprehensive economic evaluation of the telerehabilitation system, which complements the clinical analyses and addresses a critical gap in MS rehabilitation research. A cost-utility analysis will be conducted to compare the telerehabilitation intervention and usual care, using incremental cost-effectiveness ratios derived from health care service use data and EQ-5D-5L–based quality-adjusted life years. Cost data will include implementation, infrastructure, maintenance, training, support services, and any differences in health service use or patient-reported outcomes. These data will be collected alongside the RCT to ensure alignment between clinical effectiveness and economic impact.

### Ethical Considerations

Ethical approval has been obtained for WP2 from the Sheba Institutional Review Board (IRB; SMC-1154-24; May 1, 2024); the University of Limerick Education and Health Sciences Research Ethics Committee (2025_01_17_EHS; May 11, 2025); and the Central Denmark Region Committees on Health Research Ethics (1-10-72-140-24). Approval of the ethics application for WP3 is expected by September 2025. Ethics applications for the remaining WPs, including the multicenter feasibility RCT (WP4) and the economic evaluation (WP5), will be submitted to the appropriate IRB in each participating country prior to their initiation.

For WP2, all participants provide written informed consent prior to enrollment. The consent process includes comprehensive information on the study procedures, risks, benefits, and data confidentiality. All collected data are deidentified prior to analysis and securely stored in accordance with institutional data protection policies. Participants in WP2 do not receive financial compensation but may be reimbursed for travel expenses when applicable. Compensation for WP3 and future phases will be determined and approved through local IRB processes. No identifiable images or personal information will be included in the manuscript or any supplementary materials. If such materials are necessary for dissemination, explicit written consent will be obtained and submitted as required.

## Results

The PLATINUMS project received official funding in February 2024. Data collection for WP2 (the system usability and feasibility study) began in April 2025 across participating sites in Israel, Italy, Ireland, and Denmark. WP3 (validation of remote mobility measures) is scheduled to launch in September 2025, and the multicenter RCT (WP4) is planned to begin in January 2026. Project coordination and dissemination activities (WP7) have been ongoing since March 2025. Initial findings from WP2 are expected to be available by October 2025, with preliminary publications planned for mid-2026.

## Discussion

The PLATINUMS project aims to evaluate an innovative AI-based telerehabilitation system for people with MS, and if successful, it is expected to significantly advance the way rehabilitation is delivered to this population. The system is designed to provide accessible, individualized exercise programs with real-time feedback and therapist supervision, thereby overcoming some of the longstanding barriers to facility-based care. Through its structured, multiphase approach, the project will generate essential data on usability, feasibility, clinical validity of remote assessments, and preliminary efficacy of telerehabilitation interventions.

This protocol builds on a growing body of research demonstrating the potential of telerehabilitation to support physical function in people with MS while addressing several limitations observed in earlier studies. A recent scoping review identified two main categories of telerehabilitation interventions, exergaming and web-based physical therapy, both of which have shown promising results in improving gait and balance in people with MS [49]. For example, exergaming systems such as Microsoft Kinect–based virtual reality and home-based balance training tools led to significant improvements in ambulation speed, dynamic balance, and postural control [50,51]. Similarly, web-based physical therapy programs, including both those with real-time supervised and asynchronous formats, have demonstrated improvements in gait function and balance scores [52,53].

While previous interventions often lacked scalability or relied on specialized hardware, the PLATINUMS system leverages AI and widely available consumer devices, making it more practical for widespread use. The planned international feasibility trial further enhances the study’s relevance by including participants across diverse health care contexts, thereby increasing the potential for future implementation.

A particular strength of this study is its integration of technological innovation with a rigorous clinical and economic evaluation. By examining feasibility alongside psychometric testing and cost-effectiveness, the project will provide comprehensive insights into the practicality of scaling up such solutions in real-world settings. The inclusion of both patient-centered outcomes and system-level data will allow for a nuanced understanding of how AI-driven telerehabilitation can influence care delivery for people with MS. Still, as this is a protocol, all conclusions must remain provisional until data collection is complete. It will be important to interpret future findings with an understanding of the feasibility trial’s exploratory nature.

Several limitations and potential challenges should be acknowledged. First, as a feasibility study, the sample size may not be sufficient to detect small but clinically meaningful effects, and findings may not be generalizable beyond the populations and settings involved. Second, adherence to home-based interventions can be variable and difficult to monitor objectively outside controlled settings. To address this, we will incorporate remote use tracking, patient engagement strategies, and therapist follow-up. Third, technical issues such as hardware compatibility, internet connectivity, and user literacy may affect intervention delivery. These will be documented throughout the study and considered in the interpretation of results. Importantly, as with any AI-enabled solution, algorithm transparency, how well it can adapt to individual needs, and potential biases must be critically evaluated before broader deployment. Given that this is a feasibility protocol, any conclusions will remain provisional until the trial is completed and the data are evaluated.

Looking forward, the outcomes of this study are expected to inform the design of a fully powered RCT, as well as broader efforts to integrate digital health technologies into routine clinical practice. The knowledge generated may also prove relevant to other neurological conditions where mobility impairments are prevalent. A dedicated dissemination strategy has been developed to ensure that findings reach key stakeholders, including clinicians, patients, and health policy decision-makers. Ultimately, the PLATINUMS project seeks not only to assess a novel intervention but to lay the groundwork for a more accessible, personalized, and sustainable model of rehabilitation for people with MS.

## Appendix

Multimedia Appendix 1 SPIRIT (Standard Protocol Items: Recommendations for Interventional Trials) checklist.

Multimedia Appendix 2 Peer-review report from the funding agency (THCS Call Steering Committee; European Partnership on Transforming Health and Care Systems (THCS), Horizon Europe, European Union.

## Abbreviations

20SLS: 20-second single-leg stance
30STS: 30sec-sit-to-stand test
6MWT: 6-minute walk test
AI: artificial intelligence
EDSS: Expanded Disability Status Scale
FITT-VP: frequency, intensity, time, type, volume, and progression
FRT: functional reach test
IRB: institutional review board
MFIS: Modified Fatigue Impact Scale
MS: multiple sclerosis
MSIS-29: 29-item Multiple Sclerosis Impact Scale
MSWS-12: 12-item Multiple Sclerosis Walking Scale
PLATINUMS: Implementation of an Advanced Telerehabilitation Solution for People With Multiple Sclerosis
QIDS: Quick Inventory of Depression Symptomology
RCT: randomized controlled trial
RQ: research question
SDMT: Symbol Digit Modality Test
SPPB: Short Physical Performance Battery
STAI: State-Trait Anxiety Inventory
T25FWT: timed 25-foot walk test
TUG: timed up and go test
TUQ: Telehealth Usability Questionnaire
WP: working package

## Data Availability

Data sharing is not applicable at this stage as the PLATINUMS project is currently in progress. Data collection for WP2 is ongoing, and WP3 is set to commence in September 2025. Once data collection and analysis are complete, deidentified datasets may be made available upon reasonable request and subject to ethical approval and data protection regulations. Future datasets will be considered for deposition in a publicly accessible repository in accordance with Horizon Europe open science guidelines and JMIR’s data sharing policy.

## References

[1] Walton C, King R, Rechtman L, Kaye W, Leray E, Marrie RA, Robertson N, La Rocca N, Uitdehaag B, van der Mei I, Wallin M, Helme A, Angood Napier C, Rijke N, Baneke P (2020). Rising prevalence of multiple sclerosis worldwide: insights from the Atlas of MS, third edition. Mult Scler.

[2] Heesen C, Böhm J, Reich C, Kasper J, Goebel M, Gold S (2008). Patient perception of bodily functions in multiple sclerosis: gait and visual function are the most valuable. Mult Scler.

[3] Green R, Cutter G, Friendly M, Kister I (2017). Which symptoms contribute the most to patients' perception of health in multiple sclerosis?. Mult Scler J Exp Transl Clin.

[4] van der Feen F, de Haan G, van der Lijn I, Heersema D, Meilof J, Heutink J (2020). Independent outdoor mobility of persons with multiple sclerosis - A systematic review. Mult Scler Relat Disord.

[5] Kalron A, Dvir Z, Achiron A (2010). Walking while talking--difficulties incurred during the initial stages of multiple sclerosis disease process. Gait Posture.

[6] Skjerbæk Anders G, Dalgas U, Stenager E, Boesen F, Hvid LG (2023). The six spot step test is superior in detecting walking capacity impairments compared to short- and long-distance walk tests in persons with multiple sclerosis. Mult Scler J Exp Transl Clin.

[7] Souza A, Kelleher A, Cooper R, Cooper RA, Iezzoni LI, Collins DM (2010). Multiple sclerosis and mobility-related assistive technology: systematic review of literature. J Rehabil Res Dev.

[8] Zanotto T, Sosnoff JJ, Backus D, Yarnot R, Worikat NA, Abou L, Peterson EW, Rice LA (2023). Characteristics and consequences of falls among people with multiple sclerosis who use wheelchairs or scooters: Differences between injurious and non-injurious falls. Mult Scler Relat Disord.

[9] Andersen TM, Andersen AM, Riemenschneider M, Taul-Madsen L, Diechmann M, Gaemelke T, Dalgas U, Brønd Jan Christian, Hvid LG (2025). Comprehensive evaluation of accelerometer-based physical activity in persons with multiple sclerosis - The influence of disability status and its impact on walking capacity. Mult Scler Relat Disord.

[10] Sandroff B, Klaren R, Motl R (2015). Relationships among physical inactivity, deconditioning, and walking impairment in persons with multiple sclerosis. J Neurol Phys Ther.

[11] Motl R (2010). Physical activity and irreversible disability in multiple sclerosis. Exerc Sport Sci Rev.

[12] Schmidt S, Jöstingmeyer Petra (2019). Depression, fatigue and disability are independently associated with quality of life in patients with multiple sclerosis: results of a cross-sectional study. Mult Scler Relat Disord.

[13] Naci H, Fleurence R, Birt J, Duhig A (2010). The impact of increasing neurological disability of multiple sclerosis on health utilities: a systematic review of the literature. J Med Econ.

[14] Kobelt G, Eriksson J, Phillips G, Berg J (2017). The burden of multiple sclerosis 2015: Methods of data collection, assessment and analysis of costs, quality of life and symptoms. Mult Scler.

[15] Dalgas U, Langeskov-Christensen M, Stenager E, Riemenschneider M, Hvid LG (2019). Exercise as medicine in multiple sclerosis-time for a paradigm shift: preventive, symptomatic, and disease-modifying aspects and perspectives. Curr Neurol Neurosci Rep.

[16] Motl RW, Sandroff BM (2015). Benefits of exercise training in multiple sclerosis. Curr Neurol Neurosci Rep.

[17] Latimer-Cheung AE, Pilutti LA, Hicks AL, Martin Ginis KA, Fenuta AM, MacKibbon KA, Motl RW (2013). Effects of exercise training on fitness, mobility, fatigue, and health-related quality of life among adults with multiple sclerosis: a systematic review to inform guideline development. Arch Phys Med Rehabil.

[18] Casey B, Coote S, Shirazipour C, Hannigan A, Motl R, Martin Ginis K, Latimer-Cheung A (2017). Modifiable psychosocial constructs associated with physical activity participation in people with multiple sclerosis: a systematic review and meta-analysis. Arch Phys Med Rehabil.

[19] Bozkurt T, Unal M, Salci Y (2025). Factors influencing adherence to physical exercise in patients with multiple sclerosis: a systematic review focusing on exercise over general physical activity. Acta Neurol Belg.

[20] Correale L, Martinis L, Tavazzi E, Pedullà Ludovico, Mallucci G, Brichetto G, Bove M, Ponzio M, Borrelli P, Monti MC, Bergamaschi R, Montomoli C (2022). Barriers to exercise and the role of general practitioner: a cross-sectional survey among people with multiple sclerosis. Front Neurol.

[21] Moumdjian L, Smedal T, Arntzen EC, van der Linden ML, Learmonth Y, Pedullà Ludovico, Tacchino A, Novotna K, Kalron A, Yazgan YZ, Nedeljkovic U, Kos D, Jonsdottir J, Santoyo-Medina C, Coote S (2022). Impact of the COVID-19 pandemic on physical activity and associated technology use in persons with multiple sclerosis: an international RIMS-SIG mobility survey study. Arch Phys Med Rehabil.

[22] Pedullà Ludovico, Santoyo-Medina Carme, Novotna Klara, Moumdjian Lousin, Smedal Tori, Arntzen Ellen Christin, van der Linden Marietta L, Learmonth Yvonne, Kalron Alon, Güngör Feray, Nedeljkovic Una, Kos Daphne, Jonsdottir Johanna, Coote Susan, Tacchino Andrea (2023). Physical activity in multiple sclerosis: meeting the guidelines at the time of the COVID-19 pandemic. J Neurol Phys Ther.

[23] Jonsdottir J, Santoyo-Medina C, Kahraman T, Kalron A, Rasova K, Moumdjian L, Coote S, Tacchino A, Grange E, Smedal T, Arntzen EC, Learmonth Y, Pedulla L, Quinn G, Kos D (2023). Changes in physiotherapy services and use of technology for people with multiple sclerosis during the COVID-19 pandemic. Mult Scler Relat Disord.

[24] Truijen S, Abdullahi A, Bijsterbosch D, van Zoest E, Conijn M, Wang Y, Struyf N, Saeys W (2022). Effect of home-based virtual reality training and telerehabilitation on balance in individuals with Parkinson disease, multiple sclerosis, and stroke: a systematic review and meta-analysis. Neurol Sci.

[25] Ghahfarrokhi MM, Banitalebi E, Negaresh R, Motl RW (2021). Home-based exercise training in multiple sclerosis: a systematic review with implications for future research. Mult Scler Relat Disord.

[26] Najafi P, Motl RW, Moghadasi M (2025). Tele-exercise in multiple sclerosis: Systematic review and meta-analysis of effects on fatigue, depression, and overall health. Mult Scler Relat Disord.

[27] Buckingham SA, Sein K, Anil K, Demain S, Gunn H, Jones RB, Kent B, Logan A, Marsden J, Playford ED, Freeman J (2022). Telerehabilitation for physical disabilities and movement impairment: a service evaluation in South West England. J Eval Clin Pract.

[28] WizeCare - Digital Health Platform. WizeCare.

[29] Tea F, Groh AMR, Lacey C, Fakolade A (2024). A scoping review assessing the usability of digital health technologies targeting people with multiple sclerosis. NPJ Digit Med.

[30] Thompson AJ, Banwell BL, Barkhof F, Carroll WM, Coetzee T, Comi G, Correale J, Fazekas F, Filippi M, Freedman MS, Fujihara K, Galetta SL, Hartung HP, Kappos L, Lublin FD, Marrie RA, Miller AE, Miller DH, Montalban X, Mowry EM, Sorensen PS, Tintoré Mar, Traboulsee AL, Trojano M, Uitdehaag BMJ, Vukusic S, Waubant E, Weinshenker BG, Reingold SC, Cohen JA (2018). Diagnosis of multiple sclerosis: 2017 revisions of the McDonald criteria. Lancet Neurol.

[31] Doré Benjamin, Gaudreault A, Everard G, Ayena JC, Abboud A, Robitaille N, Batcho CS (2023). Acceptability, feasibility, and effectiveness of immersive virtual technologies to promote exercise in older adults: a systematic review and meta-analysis. Sensors (Basel).

[32] Parmanto B, Lewis Allen Nelson, Graham KM, Bertolet MH (2016). Development of the Telehealth Usability Questionnaire (TUQ). Int J Telerehabil.

[33] Whitehead AL, Julious SA, Cooper CL, Campbell MJ (2016). Estimating the sample size for a pilot randomised trial to minimise the overall trial sample size for the external pilot and main trial for a continuous outcome variable. Stat Methods Med Res.

[34] Latimer-Cheung AE, Martin Ginis KA, Hicks AL, Motl RW, Pilutti LA, Duggan M, Wheeler G, Persad R, Smith KM (2013). Development of evidence-informed physical activity guidelines for adults with multiple sclerosis. Arch Phys Med Rehabil.

[35] American College of Sports Medicine (2017). ACSM's Guidelines for Exercise Testing and Prescription, 10th Edition.

[36] Nilsagård Ylva, Andreasson M, Carling A, Vesterlin H (2017). Examining the validity and sensitivity to change of the 5 and 10 sit-to-stand tests in people with multiple sclerosis. Physiother Res Int.

[37] Motl R, Chaparro G, Hernandez M, Balto J, Sandroff B (2018). Physical function in older adults with multiple sclerosis: an application of the Short Physical Performance Battery. J Geriatr Phys Ther.

[38] Baert I, Freeman J, Smedal T, Dalgas U, Romberg A, Kalron A, Conyers H, Elorriaga I, Gebara B, Gumse J, Heric A, Jensen E, Jones K, Knuts K, Maertens de Noordhout B, Martic A, Normann B, Eijnde BO, Rasova K, Santoyo Medina C, Truyens V, Wens I, Feys P (2014). Responsiveness and clinically meaningful improvement, according to disability level, of five walking measures after rehabilitation in multiple sclerosis: a European multicenter study. Neurorehabil Neural Repair.

[39] Polman CH, Rudick RA (2010). The multiple sclerosis functional composite: a clinically meaningful measure of disability. Neurology.

[40] Franchignoni F, Horak F, Godi M, Nardone A, Giordano A (2010). Using psychometric techniques to improve the Balance Evaluation Systems Test: the mini-BESTest. J Rehabil Med.

[41] Soke F, Eldemir S, Ozkan T, Ozkul C, Ozcan Gulsen E, Gulsen C, Eldemir K, Irkec C, Bilge Gonenli K, Batur Caglayan HZ, Guclu-Gunduz A (2022). The functional reach test in people with multiple sclerosis: a reliability and validity study. Physiother Theory Pract.

[42] McGuigan C, Hutchinson M (2004). Confirming the validity and responsiveness of the Multiple Sclerosis Walking Scale-12 (MSWS-12). Neurology.

[43] Spielberger C, Gorsuch R, Lushene R (1983). Manual for State-Trait Anxiety Inventory (Form Y) Self-Evaluation Questionnaire.

[44] Rush A, Trivedi MH, Ibrahim HM, Carmody TJ, Arnow B, Klein DN, Markowitz JC, Ninan PT, Kornstein S, Manber R, Thase ME, Kocsis JH, Keller MB (2003). The 16-Item Quick Inventory of Depressive Symptomatology (QIDS), clinician rating (QIDS-C), and self-report (QIDS-SR): a psychometric evaluation in patients with chronic major depression. Biol Psychiatry.

[45] Campbell JA, Ahmad H, Chen G, van der Mei I, Taylor BV, Claflin S, Henson GJ, Simpson-Yap S, Laslett LL, Hawkes K, Hurst C, Waugh H, Palmer AJ (2023). Validation of the EQ-5D-5L and psychosocial bolt-ons in a large cohort of people living with multiple sclerosis in Australia. Qual Life Res.

[46] Riazi A, Hobart J, Lamping D, Fitzpatrick R, Thompson A (2002). Multiple Sclerosis Impact Scale (MSIS-29): reliability and validity in hospital based samples. J Neurol Neurosurg Psychiatry.

[47] Kos D, Kerckhofs E, Carrea I, Verza R, Ramos M, Jansa J (2005). Evaluation of the Modified Fatigue Impact Scale in four different European countries. Mult Scler.

[48] Strober L, DeLuca J, Benedict RH, Jacobs A, Cohen JA, Chiaravalloti N, Hudson LD, Rudick RA, LaRocca NG, Multiple Sclerosis Outcome Assessments Consortium (MSOAC) (2019). Symbol Digit Modalities Test: a valid clinical trial endpoint for measuring cognition in multiple sclerosis. Mult Scler.

[49] Doherty F, Powell P, McBride C, Monaghan K (2024). Physical telerehabilitation interventions for gait and balance in multiple sclerosis: a scoping review. J Neurol Sci.

[50] Ortiz-Gutiérrez Rosa, Cano-de-la-Cuerda R, Galán-del-Río Fernando, Alguacil-Diego I, Palacios-Ceña Domingo, Miangolarra-Page J (2013). A telerehabilitation program improves postural control in multiple sclerosis patients: a Spanish preliminary study. Int J Environ Res Public Health.

[51] Pagliari C, Di Tella S, Jonsdottir J, Mendozzi L, Rovaris M, De Icco R, Milanesi T, Federico S, Agostini M, Goffredo M, Pellicciari L, Franceschini M, Cimino V, Bramanti P, Baglio F (2024). Effects of home-based virtual reality telerehabilitation system in people with multiple sclerosis: a randomized controlled trial. J Telemed Telecare.

[52] Fjeldstad-Pardo C, Thiessen A, Pardo G (2018). Telerehabilitation in multiple sclerosis: results of a randomized feasibility and efficacy pilot study. Int J Telerehabil.

[53] Eldemir K, Guclu-Gunduz A, Eldemir S, Saygili F, Ozkul C, Irkec C (2024). Effects of Pilates-based telerehabilitation on physical performance and quality of life in patients with multiple sclerosis. Disabil Rehabil.

