# Supplementary material for: An AI-Based Telerehabilitation Solution to Improve Mobility in People With Multiple Sclerosis (the PLATINUMS Project): Protocol for an Implementation and Evaluation Study
Source: JMIR Res Protoc. 2025 Jul 24;14:e75983. doi: 10.2196/75983 (PMC12332452; doi:10.2196/75983)
Supplement: Multimedia Appendix 2 [file resprot_v14i1e75983_app2.pdf]

Transforming Health & Care Systems Programme

Evaluation Summary Report (ESR)

Evaluation call 2023

Proposal: Implementation of an advanced telerehabilitation solution for people with multiple sclerosis

Nr. 1538

Excellence

- Relevance:

The proposal is highly relevant to the aim and the scope of the call. The patient-centered proposed intervention can yield substantial benefits for both the present and future of health care systems and for the society as a whole.

- The quality of the proposed R&I activities:

Overall the project objectives are clearly define. The description of R&I in this project is unambiguous and easy to follow. A major weakness with the project is that the team does not consider potential barriers to exercise and the unintended consequences of the project on inequalities.

Impact

- Potential impact of the proposed research and innovation:

If successful, the project will establish a telerehabilitation program equipped with AI-based feedback, specifically tailored for multiple sclerosis patients. The research design is adequate, convincing and credible. The research includes perspectives from different disciplines and justify very well this point. The project is likely to have a high impact on the dimensions considered as key in the call, even if the innovation is not so pathbreaking

- Communication, engagement and exploitation:

There's a clear communication strategy targeting healthcare professionals and patient communities. However, the communication plan appears more oriented towards specialists rather than the general public or patients directly.

Implementation

- The quality of the project coordinator and project consortium:

The consortium is composed of foremost MS rehabilitation specialists from their respective countries, who have showcased not only significant scientific contributions on this topic but also consistent dedication in their daily clinical practice. Weakness is a lack of health economics. Gender imbalance within the team.

Co-funded by the European Union

- The quality of the project organization and management

The proposal does an excellent work describing the steps due to the project implementation. The organisation seems credible and convincing, the budget is adequate and the distribution of tasks, reasonable.

Recommendations

- Possible recommendations for future applications

This is a compelling proposal that stands out due to its clarity in writing and formulation. The dedication and expertise of the consortium members are evident, with each being leading specialists in their domain. The proposal's organization, approach, and methods are well-articulated, making it easy to grasp the intent and objectives.

Result: Grade B+ The proposal has been assessed as fairly good to good quality. Therefore it is recommended for funding.

Co-funded by the
European Union
